# Genome Sequence of the Petroleum Hydrocarbon-Degrading Bacterium *Alcanivorax* sp. Strain 97CO-5

**DOI:** 10.1128/genomeA.01277-14

**Published:** 2014-12-11

**Authors:** Xiao Luan, Zhisong Cui, Wei Gao, Qian Li, Xiaofei Yin, Li Zheng

**Affiliations:** Marine Ecology Research Center, the First Institute of Oceanography, State Oceanic Administration of China, Qingdao, People’s Republic of China

## Abstract

*Alcanivorax* sp. strain 97CO-5 was isolated from a crude-oil-degrading consortium, enriched from Yellow Sea sediment of China. Here, we present the draft genome of strain 97CO-5, which comprises 3,251,558 bp with a G+C content of 54.54% and contains 2,962 protein-coding genes and 42 tRNAs.

## GENOME ANNOUNCEMENT

The bacteria of genus *Alcanivorax* are recognized as typical representatives of obligate hydrocarbonoclastic bacteria (OHCB), which play a pivotal role in the biological removal of petroleum hydrocarbons from polluted marine environments (1–3). *Alcanivorax* sp. strain 97CO-5 (CGMCC no. 3735) was isolated from Yellow Sea sediment of China (lat 36.67; long 121.99; depth 17.8 m) for its ability to use *n*-alkanes as a sole carbon and energy source (4). Many genome sequences of the *Alcanivorax* species have been published since the report of the first sequenced genome of *Alcanivorax borkumensis* SK2 (GenBank accession number AM286690), which is a ubiquitous hydrocarbon-degrading marine bacterium (3). Nevertheless, a genome sequence of an alkane-degrading species *Alcanivorax* sp. 97CO-5 from Yellow Sea sediment of China could further aid in understanding the *Alcanivorax* species’ biodiversity and capacity for petroleum hydrocarbon biodegradation in marine environments.

The genome sequence of *Alcanivorax* sp. 97CO-5 was determined by Shanghai Majorbio Bio-pharm Technology Co., Ltd. (Shanghai, China) using Solexa paired-end sequencing technology. A total of 16,700,406 paired-end reads (180-bp and 800-bp libraries) were generated with the Illumina HiSeq 2000 platform (Illumina Inc., SanDiego, CA, USA) to reach a 513-fold coverage. The reads were assembled using SOAPdenovo version 1.05 (5, 6). The resulting genome sequence of *Alcanivorax* sp. 97CO-5 consists of 37 contigs (*N*_90_ = 79,842) of 3,251,558 bp with an average G+C content of 54.54%. Gene annotation was carried out using the NCBI Prokaryotic Genomes Automatic Annotation Pipeline (PGAAP) (http://www.ncbi.nlm.nih.gov/genomes/static/Pipeline.html), which was followed by manual editing. The genome contains 2,962 candidate protein-encoding genes (with an average size of 959 bp), giving a coding intensity of 87.40%. A total of 2,192 proteins were assigned to cluster of orthologous groups (COG) families, and 42 tRNA genes for all 20 amino acids and one 16S-23S-5S rRNA operon were identified by tRNAscan version 1.23 (7) and rRNAmmer version 1.2 (8).

In particular, we analyzed the genes possibly responsible for alkane degradation. Two genes encoding alkane 1-monooxygenase and two genes encoding cytochrome P450 enzymes were found in the genome of *Alcanivorax* sp. 97CO-5. Moreover, one gene encoding the haloalkane dehalogenase was also found in the genome sequence. The 97CO-5 genome sequence and its curated annotation are important assets for better understanding the physiology and metabolic potential of *Alcanivorax* spp. in the Yellow Sea and will open up new opportunities in the functional genomics of this species.

### Nucleotide sequence accession number.

The nucleotide sequence comprising the *Alcanivorax* sp. 97CO-5 genome was deposited at DDBJ/EMBL/GenBank under the accession number AZYR00000000 (chromosome). The version described in this paper is the first version.
